# Glances at the Hospitals

**Published:** 1900-05-19

**Authors:** 


### GLANCES AT THE HOSPITALS.

### THE DEVONSHIRE HOSPITAL AT BUXTON.

The mineral waters of Buxton have been celebrated for centuries in the treatment of rheumatic affections, and as early as the year 1572 Dr. Jones advised those fortunate people who had money to spare to give a little to those who had none, so that the latter might participate in the benefits derived from the treatment. Money was collected for this charitable purpose, and the fund was known as the "Treasury of the Bath." In 1785 a document, apparently an annual report of the institution, was brought to light, and is now carefully preserved in the hospital archives. The present Buxton Bath Charity and Hospital is intended for the relief of poor persons suffering from rheumatism, gout, sciatica, paralysis, dyspepsia, uterine disease, &c., and when it is stated that during 1899 there were 2,835 in-patients under treatment, it will be seen that the institution has a wide influence for good; 2,485 patients were discharged improved, 196 not improved, 6 as unsuitable for treatment, 22 at their own request, 9 for drunkenness, and 14 for various other causes. There were 7 deaths, and 96 remained on the books. As patients are admitted from all parts of Great Britain and Ireland, it is not to be wondered at that many find it difficult to pay their travelling expenses when the distance is great. The Charity Organisation Society have given help in this direction, and there is a Samaritan Fund connected with the hospital which pays the whole or part of the railway fares of needy patients. The managers would gladly receive subscriptions for this fund. The out-patients numbered 201, and of these 139 were discharged improved, 12 not improved, and 44 left without report, and five remained under treatment at the end of the year. On discharge each patient is given a postcard, and this should be sent back by the subscribers within the year, so that the medical staff can arrive at a better estimate of the benefit derived by the patient. The average daily number of patients was 172. January was the lowest month with 62, and September the highest with 257. Like other hospitals, this charity derives its income from annual subscriptions, casual subscriptions, donations, and from collections made in churches and hotels.

### NORTHALLERTON COTTAGE HOSPITAL.

The year's working of this hospital shows that it had 5 patients under treatment on January 1st, 1899; that 98 were admitted during the year, that 2 died, and that 9 remained under treatment. The latter is about the average daily number in residence, and each patient spent an average of 34 days in the hospital. The pecuniary position of the hospital is not wholly satisfactory, as there was a deficit of £70 on the general account, and yet there does not seem to be any extravagance in the management, so that the fault lies in the smallness of the annual subscriptions. Last year there was a slight increase in the subscriptions and also in the payments made by patients, the total of the former amounting to about £533, and the latter to £147. The managers record their appreciation of the services of Miss Atkinson, the matron.

### THE DEWSBURY GENERAL INFIRMARY.

This hospital was established in 1876, and it has just issued its twenty-fourth annual report. The total income amounted to £2,236, being made up of £413 in subscriptions, £153 in donations; Hospital Saturday produced £1,095, and Hospital Sunday £166. The rest was made up of rent, sales, interest, and special subscriptions to the Children's Ward Fund. The total expenditure was £2,121. Both income and expenditure were slightly above those of the year 1898, and the board foreshadows that for 1900 the expenditure will be still higher, because the number of patients will increase and certain structural alterations should be carried out. Last year there were 477 in-patients and 2,547 out-patients; the former number shows an increase of 37, and the latter a decrease of 667. Of the in-patients, 416 were discharged cured or relieved, 6 were discharged for other reasons, 24 died, and 31 remained in the hospital at the end of the year; 287 operations were performed, and 1,178 minor accidents were treated in the casualty ward. The children's ward dates from 1897 only, and has proved a boon to a large number of children; but, unfortunately, it has not been a financial success, as the total income of this department stopped short at £28 14s. 9d. Dr. Prior resigned the office of house surgeon, which he had held for four years, and the board pay a tribute to his services in the hospital. Dr. Faull was appointed his successor. The domestic and nursing departments are under the management of the matron, Miss E. Nunn, and the managers say that both departments reflect great credit on her and her staff. She has under her four nurses and two probationers. During the twenty-four years of its existence 83,506 patients have availed themselves of the benefits of the charity, a number showing at once the popularity of the hospital, and the enormous amount of gratuitous work done by the medical men in a town of thirty thousand inhabitants.

